# Simplifying Laparoscopic Nephrectomy for Beginners: Double Window Technique With En Bloc Hilar Stapling

**DOI:** 10.7759/cureus.16090

**Published:** 2021-07-01

**Authors:** Tarun Jindal, Satyadip Mukherjee, Rajan Koju, Nitesh S, Denchu Phom

**Affiliations:** 1 Urology, Tata Medical Center, Kolkata, IND; 2 Urology, Tata Medical center, Kolkata, IND

**Keywords:** laparoscopy, kidney, nephrectomy, hilum, staplers, safe surgery

## Abstract

Laparoscopic nephrectomy is a commonly performed procedure. As with any surgical procedure, this too has a significant learning curve. The management of renal hilum is the most critical part of this surgery. It requires a meticulous intra-hilar dissection to identify the renal artery and vein. The kidneys are extremely vascular structures and any injury to these vessels during dissection can result in life-threatening bleeding. Hence, it is obvious that beginners most often face difficulty and apprehension at this step of the laparoscopic nephrectomy. We describe a simple technique of laparoscopic nephrectomy which includes the creation of two windows, one at the lower pole and the second at the upper pole, isolation of the hilum, and en bloc stapling of the renal hilar vessels. This method safeguards against collateral damage to the surrounding structures. It also avoids the need for intra-hilar dissection, hence decreasing the chances of vascular injuries.

## Introduction

Laparoscopic nephrectomy is a commonly performed procedure. The technique is well established and replicates the steps of open surgery. The conventional method is the inferior approach in which the lower pole is mobilized first followed by the dissection of the hilum, isolation followed by ligation of artery and vein, and then the upper and lateral mobilization [1-3]. The most critical part of laparoscopic nephrectomy is the management of the renal hilum [2,4,5]. It entails the identification and meticulous dissection of the renal artery and vein and their ligation [1-3]. This requires a significant amount of experience and may be the most difficult step for surgeons who are in the initial phase of their learning curve [4,5]. Injury to these vessels during dissection can lead to catastrophic life-threatening consequences. We describe a simpler method of laparoscopic nephrectomy, the double window technique with en bloc stapled division of the hilum. In this technique, the operator completely mobilizes the kidney at both the poles (the double windows) before managing the renal hilum. This approach encompasses an en bloc hilar stapling and does not need an intra-hilar dissection, thus avoiding the risk of renal vascular injury, especially in the hands of beginners. The technique can be used for both, simple as well as radical laparoscopic nephrectomy.

## Technical report

Instruments needed

Ports of size 5 mm, 10 mm, and 12 mm, 30-degree telescope, laparoscopic instruments like atraumatic bowel grasper, self-retaining toothed grasper for liver retraction, Hem-o-lok clip applicator, metallic clip applicator, laparoscopic scissors, suction cannula. As per the availability, one can use a monopolar hook or ultrasound-based devices like Harmonic (Ethicon, Johnson and Johnson, USA)/Thunderbeat (Olympus, USA) or bipolar devices like Ligasure (Medtronic, USA) for dissection. Laparoscopic staplers, preferably articulating, and vascular cartridges should be readily available.

Positioning of the patient

A Foley catheter is inserted and then the patient is placed in a lateral decubitus position, 90-degree to the table. The side to be operated upon should be kept up. The patient should be placed at the edge of the operating table so that the surgeon does not face any restriction of movement of the instruments. We do not use the kidney bridge or flex the table during minimally invasive nephrectomy. The back of the patient is well supported by bolsters and backrests which are fixed to the table. The upper leg is kept straight while the lower leg is flexed at the knee. The arms are flexed and supported on armrests. It is important to position the arms cranially so that they do not hinder the instrument maneuverability. All the pressure points are well padded. It should also be ensured that the anesthetist should have free access to the tubings, electrodes, and vascular lines.

Creation of pneumoperitoneum

The open or closed methods can be used to create the pneumoperitoneum. We prefer to use the Veress needle for this step. The surgeon should check the patency of the needle ex-vivo by flushing with saline. The retractibility of the tip should also be checked before the introduction. We routinely insert the Verees needle slightly lateral to the midpoint of the spinoumbilical line. A 5 ml syringe filled with saline is attached to the needle and aspiration is done to rule out entering into a hollow viscus or a vessel. A hanging drop test can be performed to confirm the entry into the peritoneum. The gas is connected after this and a target pressure of 14 mm of mercury is attained. At this step, one should ensure that the abdomen is distending uniformly and also a watch should be kept on the smooth rise of pressure as shown in the insufflation machine.

Port placement for left side nephrectomy

**Figure 1:**
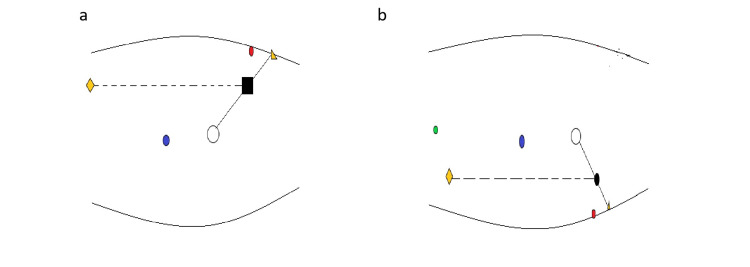
Port positioning for laparoscopic nephrectomy (a) The port positions for a left nephrectomy: 12 mm port at the spinoumbilical line (Black box), 5 mm port in the subcostal area in line with 12 mm port (yellow quadrangle), 10 mm camera port (blue circle) and the retraction port above the anterior superior spinal process (red circle), (b) The ports for a right nephrectomy: 5 mm port at the spinoumbilical line (Black circle), 12 mm port in the subcostal area in line with 5 mm port (yellow quadrangle), 10 mm camera port (blue circle), the retraction port above the anterior superior spinal process (red circle) and a 5 mm liver retraction port in the epigastrium (Green circle)

The first port is a 12 mm port placed at the spinoumbilical line, slightly lateral to the midpoint. A 5 mm port is placed at the sub-costal area in a straight line to the 12 mm port. A 10 mm port is now placed by the triangulation method which will act as the camera port. A 5 mm port is subsequently placed at 2 cm above the anterior superior iliac spine which is used to lift the ureter during the surgery (Figure 1a).

Port placement for right side nephrectomy

A similar approach is used on the right side except that a 5 mm port is used at the spinoumbilical level and the 12 mm port is deployed at the subcostal area. A 5 mm liver retraction port is placed in the epigastric area the site of which will depend upon the size and shape of the liver (Figure 1b).

Steps of surgery

Diagnostic Laparoscopy

The first step of any laparoscopic surgery is an optimal diagnostic laparoscopy. The operating surgeon should identify all the landmarks needed for the surgery. Any abnormal deposits, conditions of the liver and spleen should be documented.

Colon Mobilization

**Figure 2:**
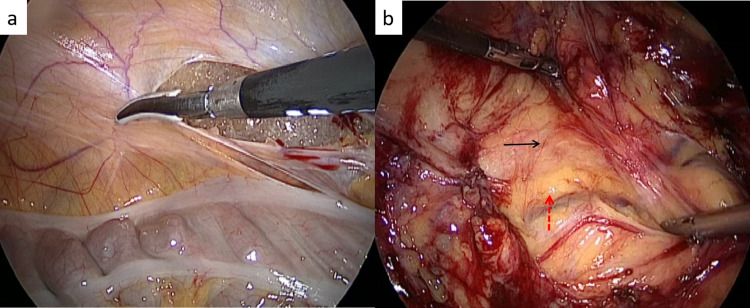
Colon mobilization (a) The peritoneum being incised to mobilize the colon, (b) The plane being created between the bright yellow mesenteric fat (broken red arrow) and the dull yellow retroperitoneal fat (black arrow)

The colon is mobilized by incising the white line of Toldt (Figure 2a). On the left side, one should start at the level of the pelvic brim and work towards the spleen while on the right side, it’s more ergonomic to start at the level of the liver and work caudally. The colon should be completely mobilized to avoid working in tunnels. The surgeon should take utmost care at this step when using energy devices to avoid inadvertent electric/thermal injury to the bowel. The bowel mesentery is bright yellow in color while the retroperitoneal fat is dull yellow (Figure 2b). This can help in the proper identification of the plane between the mesentery and the retroperitoneum. Thin pulsatile vessels belong to the mesentery and should not be divided. If there is undue bleeding, the plane is most likely wrong and needs revision. A blunt instrument like suction is very useful at this step to swipe the mesentery away. 

Identification of Ureter and Gonadal Vessel 

**Figure 3:**
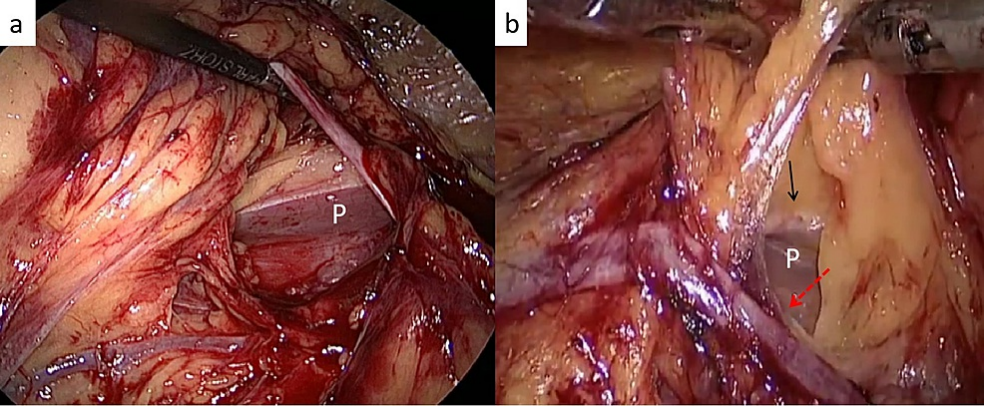
Identification of ureter and gonadal vessel (a) The left uretero-gonadal packet being lifted off the psoas muscle (P), (b) the ureter (black arrow) being separated from the gonadal vein (red arrow) on the right side. The psoas muscle (P) is also seen

The next step is to identify the psoas muscle. The surgeon should now aim to isolate the gonadal and the ureter. At times, especially early in the experience, the psoas tendon or the iliac artery may be confused with the ureter. It is important to look for the peristalsis of the ureter in case of confusion. On the left side, we prefer to lift the gonadal vessels and the ureter together while on the right side, we medialize the gonadal vessel and lift only the ureter (Figure 3a, 3b). A retractor is deployed through the 5 mm port above the iliac spine to help in lifting the ureter/uretero-gonadal packet.

Lower Pole Mobilization

**Figure 4:**
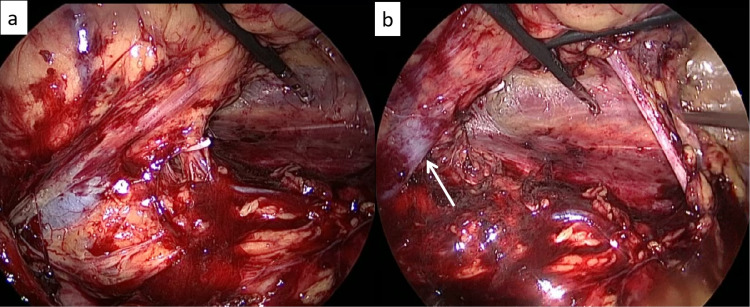
Lower pole mobilization (a) The gonadal artery has been clipped in an effort to free the lower pole of the left kidney, (b) The lower window is created with the lower margin of the renal vein (white arrow) forming the upper limit

The adventitial tissue between the ureter and the psoas is dissected with the help of blunt and sharp dissection. A few small ureteric vessels may be encountered which can be divided by the use of energy devices. The traction on the ureter is constantly re-positioned as one works towards the lower pole of the kidney. The gonadal artery is usually found just below the lower pole and can be either clipped or divided by the use of ultrasonic or bipolar energy devices (Figure 4a). The lower pole is lifted off from the psoas and at this point, the renal vein can be first visualized. We clear all the tissue below the inferior margin of the renal vein and also the lower pole of the kidney (Figure 4b). If accessory vessels to the lower pole are encountered, they can be clipped and divided. The lumbar veins can also be seen, especially on the left side, draining into the renal vein. They should be left untouched.

Upper Pole Mobilization

**Figure 5:**
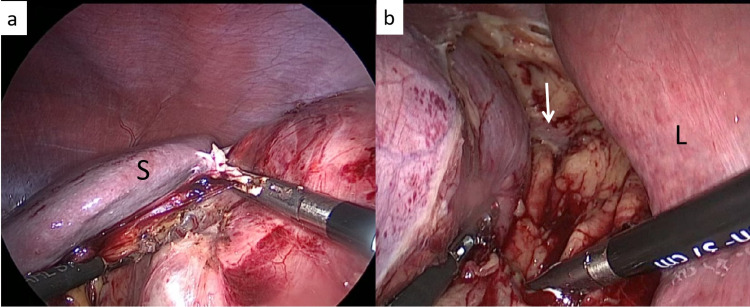
Upper pole mobilization (a) The spleno-renal ligament being divided on the left side to medialize the spleen, (b) the attachments between the liver and the upper pole of the kidney being divided on the right side. The psoas is also seen (white arrow)

The dissection now moves towards the upper pole. The spleen is mobilized completely by dividing the spleno-renal and spleno-diaphragmatic ligaments so that it falls medially (Figure 5a). On the right side, the triangular ligament may be divided to lift the liver off the upper pole. The target at this point is to see the psoas and the diaphragm behind the upper pole (Figure 5b). If adrenal is to be preserved, one can open the Gerota’s at the upper pole and work between the renal capsule and the Gerota’s fat to medialize the adrenal gland. If the adrenal gland is to be excised then space is created between the adrenal and the great vessels. The aim is to dissect all the adhesions of the upper pole and lift it off the psoas muscle. The dissection should continue medially until the upper margin of the renal vein can be well visualized. If the adrenal is to be excised, the adrenal vein is clipped and divided at this point. An accessory vessel going to the upper pole, if encountered, can also be clipped and divided. 

Creation of the Windows

**Figure 6:**
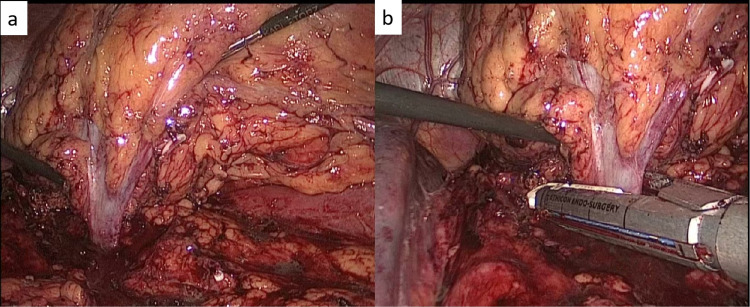
Creation of windows and hilar stapling (a) The graspers in upper and lower windows enabling the hilum to be stretched, (b) The stapler being deployed en bloc on the renal hilum. The fully medialized spleen is also seen

The upper pole and the lower pole are free of their attachments while the posterior and lateral surfaces of the kidney are still attached to the retroperitoneum. The operator should put one of the graspers at the level of the upper margin of the renal vein and another grasper below the lower margin of the vein and lift the kidney up (Figure 6a). This step helps in isolating and stretching the entire renal hilum. The camera is turned posterio-laterally and it should be ensured that no attachments are left intact behind the hilum.

Hilar Stapling

Once it is ensured that only the hilum is holding the kidney medially, a stapler loaded with a vascular stapler (white, open staple height of 2.5 mm) is introduced from the 12 mm port. The length of the cartridge, 45 mm or 60 mm, can be decided depending on the size of the renal vessels. An articulating stapler is better than a straight one as at times, the operator may have to angulate the stapler in order to ensure a good deployment. The intention is to ensure that the entire hilum is completely covered by the stapler. The hilum is kept under tension by lifting the upper and the lower poles of the kidney with two different laparoscopic graspers. A proper assessment should be done by rotating the camera all around the hilum that it has been completely covered by the cartridge. The previously created double windows make this step easy. The operator should be careful that no metal or hem-o-lok clips should be inadvertently caught between the jaws of the cartridge else it will lead to a misfire. The staple is fired after this and the hilum is divided en bloc (Figure 6b). The kidney of freed from the remaining posterior and lateral attachments and is allowed to fall medially. The ureter is clipped and divided as low as possible. The gonadal vein on the left side is divided at the level of the division of the ureter. The hemostasis in and around the renal fossa should be checked and any bleeding should be controlled. The specimen is now bagged and can be delivered out via a Lanz or Pfannenstiel incision. A drain may be placed from the 5 mm port above the iliac spine.

## Discussion

The first laparoscopic nephrectomy was performed in the early 1990s and since then it has become the standard of care in the management of kidney pathologies [6]. The distinct advantages of laparoscopic nephrectomy over its open counterpart are smaller incisions, less pain, early recovery, and decreased hospitalization time [7,8]. The technical advancements, surgical refinement, availability of better energy, and ultrasonic devices have significantly decreased the operating time and decreased the complications.

Laparoscopic nephrectomy is usually the first formal minimally invasive surgery that is taught to the residents nonetheless it is associated with a significant learning curve. It has been found that about 20-50 cases or at least a year of dedicated training at a high-volume center may be needed to overcome this learning curve [9,10]. The major challenge in the surgery is the management of the renal hilum. The kidneys are extremely vascular structures and they receive a significant part of the cardiac output. Moreover, the renal arteries and veins have a direct connection with the aorta and the inferior vena cava, respectively. The presence of the renal artery behind the renal vein also makes the identification difficult especially for beginners [11]. In a study, it was found that the majority of the complications in the early part of the learning curve happened at the time of intra-hilar dissection of the artery and vein. This ranged from injury to the main renal vein to injury to the vena cava [12]. As there is significant magnification in laparoscopic surgery, even mild bleeding can obscure the view and make the dissection very difficult. At times, this bleeding maybe even life-threatening. If there is a way that the intra-hilar dissection can be avoided, major vascular complications can be minimized. The technique of en bloc stapling obviates the need for this intra-hilar dissection and simplifies the nephrectomy for a novice. 

The double window technique also has some distinct advantages over the conventional inferior approach. In this method, the surgeon works sequentially at both the poles of the kidney and narrows down on the hilum from both sides. All the small vessels around the poles, if present, are tackled earlier than the main hilar vessels. This technique also avoids the catastrophic possibility of injury to the superior mesenteric artery as the kidney is lifted off entirely from the aorta before a stapler is deployed. It has also been shown to be easily reproducible by surgeons who are still in the early part of their experience [5]. An additional advantage of this technique is that at the time of application of the stapler, both the blades of the stapler can be well visualized as compared to the inferior approach in which the visualization of the upper blade may not be optimum.

There has been apprehension about the risk of arteriovenous fistula by the use of en bloc stapling of the renal hilum. A randomized controlled study has proved that en bloc stapling is safe and is not associated with increased long-term vascular complications. The en bloc stapling also significantly reduced the operative time and the blood loss compared to hilar dissection and clipping of the vessels [13]. As en bloc stapling does not need a hilar dissection, it can be easily performed even by beginners and the learning curve is not steep. In our experience of 44 cases of laparoscopic nephrectomies performed by this approach in the last three years, there were no stapler misfires, no cases required conversion, none of the cases had any long-term complications of a vascular fistula or cardiac failure due to en bloc stapling. The choice of stapler depends upon the operator. We have used both, Endo-GIA (Covidien-Autosuture, USA) as well as Echelon (Johnson and Johnson, USA), staplers, in our cases and there have been no issues with either of them. The 45 mm has been the most commonly used cartridge in our cases. 

We are aware that staplers are costly as compared to clips but when the question is to make the surgery safe, the cost factor becomes unimportant.

## Conclusions

To conclude, we describe a double window technique along with en bloc hilar stapling for laparoscopic nephrectomy. This technique does not need intra-hilar dissection and also decreases the chances of major vascular injuries. The technique is easy to follow and has clear steps which can be replicated. It can be extremely helpful for anyone performing laparoscopic nephrectomies for benign or malignant renal lesions, especially beginners. 
